# Outcomes of Day Care Surgery in a Tertiary Care Hospital: A Longitudinal Study

**DOI:** 10.7759/cureus.89798

**Published:** 2025-08-11

**Authors:** Sagarika S Bhole, Mohd Yunus Shah, Unmay Agarwal

**Affiliations:** 1 General Surgery, NKP Salve Institute of Medical Sciences and Research Centre, Nagpur, IND

**Keywords:** ambulatory surgery, day care surgery, keyhole surgery, outpatient clinic, outpatient hospital care, outpatient surgery

## Abstract

Background

Day care surgery offers a cost-effective and efficient alternative to inpatient care, reducing hospital bed occupancy, postoperative complications, and patient inconvenience. In India, its uptake remains limited, particularly in government hospitals. This study evaluates the feasibility, safety, outcomes, and patient satisfaction of day care surgeries in a tertiary care setting.

Methodology

A prospective, longitudinal study was conducted from November 2022 to October 2024 at a tertiary care academic hospital in Central India. In total, 86 patients, aged 1-90 years, who met the predefined inclusion criteria, underwent various minor and intermediate surgical procedures. Preoperative anesthesia clearance and informed consent were obtained. Postoperative outcomes were assessed using the ASEPSIS score and Visual Analogue Scale on postoperative days (PODs) 3, 7, and 15. Complications, readmissions, and patient satisfaction (Likert scale) were recorded. Data were analyzed using descriptive statistics and Pearson’s chi-square test using Epi Info version 7.

Results

The mean patient age was 31.24 ± 11.54 years, with a male predominance (72.94%). Spinal anesthesia (51.76%) was the most common, followed by local anesthesia (37.64%). Hernia repair (20.93%) was the most frequently performed procedure. The mean hospital stay was 20.5 ± 2.4 hours. Common complications included postoperative nausea and vomiting (25.88%) and urinary retention (3.52%). By POD 15, 83.72% of patients were pain-free, and 93.02% demonstrated satisfactory wound healing. Readmission occurred in 8.14% of cases, primarily due to urinary retention, uncontrolled pain, or vomiting. Patient satisfaction was high, with 94.12% rating the experience as satisfactory or very satisfactory.

Conclusions

Day care surgery is a safe, effective, and patient-friendly approach in a tertiary care setting, achieving low complication and readmission rates with high satisfaction. With proper patient selection and structured perioperative protocols, it can optimize resource utilization and improve surgical care delivery in resource-constrained healthcare systems.

## Introduction

Day care surgery refers to the admission and discharge of a patient on the same day for a planned surgical procedure, typically within 12 hours. While global definitions vary, with the United States considering up to a 23-hour stay as day surgery, India defines it strictly as same-day discharge [1,2]. The model offers significant benefits, including increased patient convenience, especially for children and the elderly, minimized disruption to daily life, optimized hospital bed utilization, reduced surgical cancellations, enhanced scheduling flexibility, and faster patient turnover. Clinically, it also leads to low morbidity, infection rates, and respiratory complications, while economically reducing preoperative testing, postoperative medication use, and staffing costs. Studies have shown that adopting day care surgery can lead to cost savings between 20% and 70%, mainly due to efficient use of facilities and fewer hospital bed requirements [3].

Despite these advantages, the adoption of day care surgery varies globally and nationally. The differences stem largely from disparities in insurance coverage rather than the nature of procedures. For instance, even within the same country, the proportion of procedures done as day care differs depending on whether such care is reimbursed. The United Kingdom has aggressively pushed day care surgery, with the British Association of Day Surgery aiming for 85% of elective surgeries to be done on a day care basis. The NHS also includes reforms targeting day care surgery, recommending its standardization, minimal unnecessary follow-ups, and redesigning care pathways for staff optimization. Success in the United Kingdom has been linked to dedicated resources and staff for day care surgery [4].

India faces a unique challenge. According to the 2011 census, 69% of its population resides in rural areas, but 60% of hospital beds are in the private sector, concentrated in urban zones. The government sector, which caters to rural and semi-urban populations, struggles with limited beds, fewer surgeons and anesthetists, and scarce operating rooms, all hindering the promotion of day care surgery. In contrast, private hospitals, which serve a more informed and urban demographic, show higher awareness and utilization of day care facilities [5]. As a result, only about 20% of elective surgeries in India are currently performed as day care procedures, far behind countries such as the United States, at 83.5% in 2006, or the United Kingdom, at 75% [6].

Day care surgery holds specific relevance for India’s large working population, many of whom are daily wage laborers. Traditional inpatient surgical pathways that involve three to four days of hospitalization lead to income loss. Day care surgery can address this problem by enabling quicker recovery and return to work. However, awareness in government hospitals remains low, especially low, where a significant proportion of beds is occupied by benign conditions such as gallbladder disease, appendicitis, and breast lumps, all manageable through day care protocols. Promoting day care surgery would free beds for emergency and cancer patients, reduce waiting times, and improve healthcare efficiency [7,8].

Currently, a significant research gap exists regarding the safety and outcomes of day care surgery in Central India. Establishing dedicated day care centers and conducting region-specific studies would not only improve patient outcomes but also inform health policy and resource planning, benefiting both providers and patients in the long term.

## Materials and methods

The present prospective, longitudinal study was conducted from November 2022 to October 2024 to assess the safety, outcomes, and satisfaction associated with day care surgery at a tertiary care academic hospital in Central India. It evaluated all consecutive patients who met the inclusion criteria, including those aged 1 month to 70 years, medically fit, and suitable for same-day discharge. Exclusion criteria were stringent to ensure safety, filtering out patients with uncontrolled comorbidities, high body mass index, psychiatric instability, lack of social support, or surgeries requiring more than two hours. The target sample size was 80, based on prior prevalence estimates, and eventually, 86 patients were included.

The sample size for the study was calculated based on the methodology reported by Lingaiah et al. [9]. The assumptions used for the calculation included an expected proportion (p) of 96.7%, an absolute precision (d) of ±4%, and a 95% confidence level (α) of 0.05. The following standard formula was used for sample size estimation: N = Z.Z.α .p. (100-p)/d.d, where p is the expected proportion at 96.7, d is the absolute precision, and alpha is 0.05. The required sample size was determined to be 80 participants. This calculation ensured that the study achieves the desired statistical reliability and precision in estimating proportions related to day care surgery outcomes.

Preoperative assessments included anesthesia clearance, procedure selection, and informed consent. Intraoperative monitoring was rigorous, involving the surgical and anesthetic team, with criteria such as hemorrhage, difficult airways, and excessive sedation warranting inpatient conversion. Postoperatively, patients were observed for four hours and discharged only after meeting comprehensive criteria covering vital signs, mobility, oral intake, and pain control. Follow-up occurred on days 3, 7, and 15, evaluating surgical site healing using ASEPSIS score and pain via the Visual Analog Scale score. Patient satisfaction was documented using a Likert scale, and telemedicine or WhatsApp-based communication facilitated follow-up for patients living remotely.

Data were collected and securely stored by the principal investigator, with all personal identifiers kept confidential. Entries were maintained in an Excel spreadsheet, verified weekly for accuracy, and regularly backed up. Data are presented in tabular form. Descriptive statistics, including mean and standard deviation for continuous variables and frequency with percentages for categorical variables, were calculated. Associations between categorical variables were analyzed using the Pearson chi-square test. All statistical analyses were performed using Epi Info version 7.

## Results

The demographic analysis revealed that the majority of patients were in the age group of 31-40 years (38.82%), followed by 21-30 years (25.88%), and 41-50 years (15.29%), with a mean age of 31.24 ± 11.54 years (Table 1).

**Table 1:** Age and gender distribution of study subjects.

	Female	Male	Total
1–10	-	1 (1.16%)	1 (1.16%)
11–20	1 (1.16%)	1 (1.16%)	2 (2.32%)
21–30	4 (4.65%)	18 (20.93%)	22 (25.58%)
31–40	13 (15.11%)	21 (24.41%)	34 (39.53%)
41–50	4 (4.65%)	9 (10.46%)	13 (15.11%)
51–60	-	5 (5.81%)	5 (5.81%)
61–70	1 (1.16%)	6 (6.97%)	7 (8.13%)
71–80	-	1 (1.16%)	1 (1.16%)
81–90	-	1 (1.16%)	1 (1.16%)
Total	23 (26.74%)	63 (73.25%)	86 (100%)

Males accounted for 72.94% of the study group, a statistically significant gender difference (p = 0.001), similar to findings of other Indian studies. Age and gender stratification showed that both males and females were most commonly represented in the 31-40-year age group (Table 2).

**Table 2:** Gender distribution of study subjects.

Gender	N	frequency
Male	62	72.94%
Female	24	27.90%

Spinal anesthesia was the most frequently used method (51.76%), followed by local (37.64%) and general anesthesia (10.58%). This reflects a growing trend toward regional and local anesthesia in day care surgery, as it allows quicker recovery and fewer systemic effects (Table 3).

**Table 3:** Distribution of anesthesia used.

Anesthesia	N	Frequency
General	9	10.46%
Local	33	378.37%
Spinal	44	51.16%
Total	86	100%

A wide range of procedures was performed. Hernia repair (20.93%) was the most common, aligning with global and Indian literature. This was followed by excision of lipomas (10.46%), lateral sphincterotomy (9.3%), and fibroadenoma excision and sac eversion (8.13% each). More specialized procedures, such as laparoscopic appendectomy, laparoscopic cholecystectomy, and cystoscopies, were also successfully performed, albeit in fewer patients (each 4.65% or lower) (Table 4).

**Table 4:** Distribution of procedures performed.

Procedure	N	%
Hernia repair	18	20.93
Excision of lipoma	9	10.46
Lateral sphincterotomy	8	9.3
Eversion of sac	7	8.13
Excision of fibroadenoma	7	8.13
Circumcision	5	5.81
Laparoscopic appendectomy	4	4.65
Cystoscopy	4	4.65
Excision of dermoid cyst	4	4.65
Excision of sebaceous cyst	3	3.48
Laparoscopic cholecystectomy	3	3.48
DJ stent removal	2	2.32
Inguinal lymph node biopsy	2	2.32
Arteriovenous fistula	2	2.32
Excision of epidermoid cyst	1	1.16
Hemorrhoidectomy	1	1.16
Incision and drainage of the submandibular abscess	1	1.16
Seton application in fistula in ano	1	1.16
Subcuticular mastectomy	1	1.16
Toenail excision	1	1.16
Uretroscopic lithotripsy	1	1.16
Wart excision	1	1.16
Total	86	100

Postoperative complications

Postoperative complications were minimal. The most frequently reported were nausea and vomiting (each in 25.88% of cases), while only 3.52% required urinary catheterization. These complications were transient and managed without a prolonged hospital stay.

Pain outcomes

**Figure 1:**
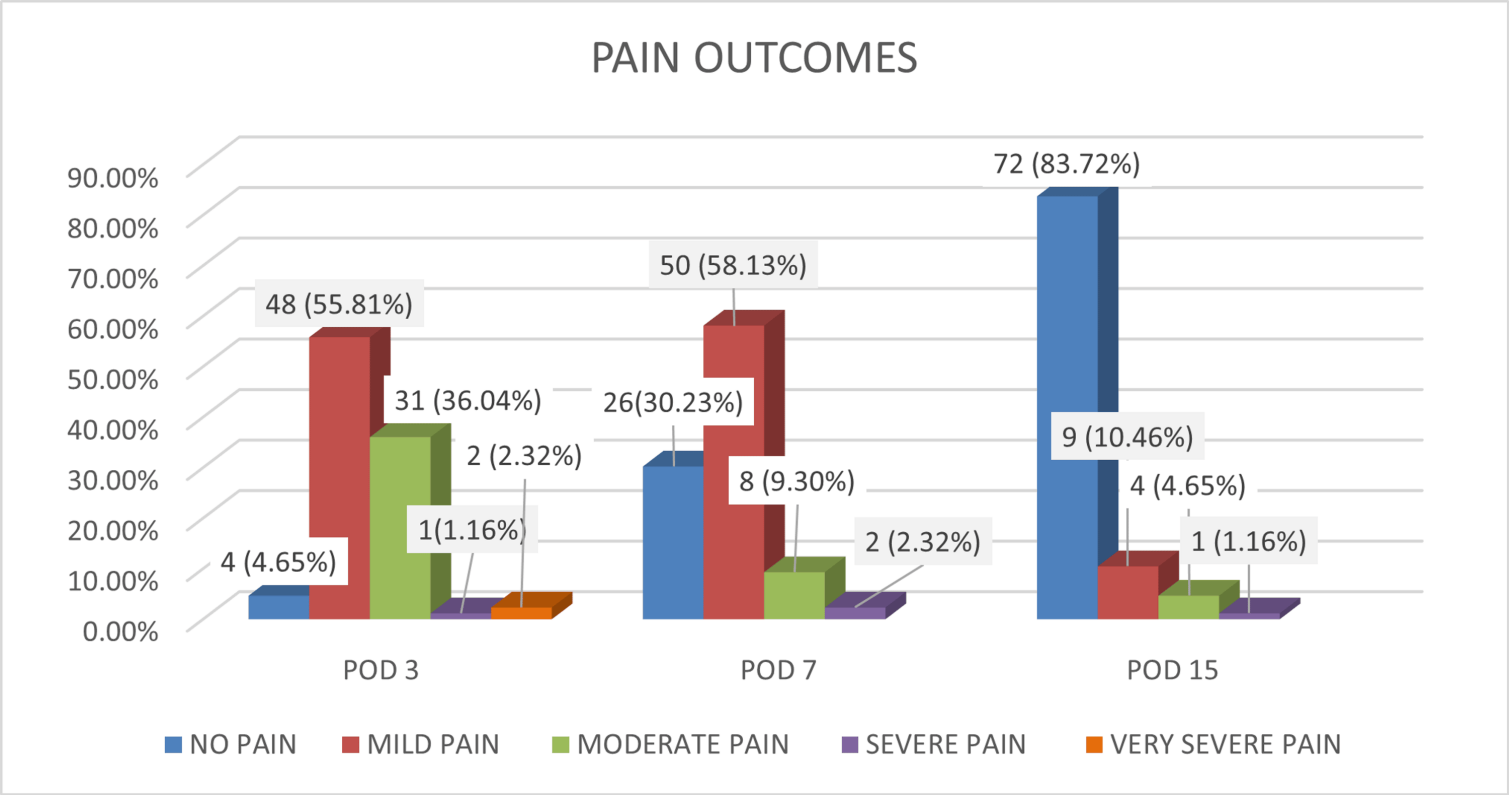
Distribution of pain outcomes (n = 86).

Pain levels decreased significantly over time. At discharge, most patients experienced moderate pain (54.3%), while by postoperative day (POD) three and seven, the majority reported mild pain (55.81% and 58.13%, respectively). By POD 15, 83.72% were pain-free. Only one patient reported high pain on POD 15 due to a sphincterotomy. The progressive reduction in mean pain scores (from 3.03 to 0.58) underscores the effectiveness of pain management protocols and patient selection criteria (Figure 1).

ASEPSIS score

**Figure 2:**
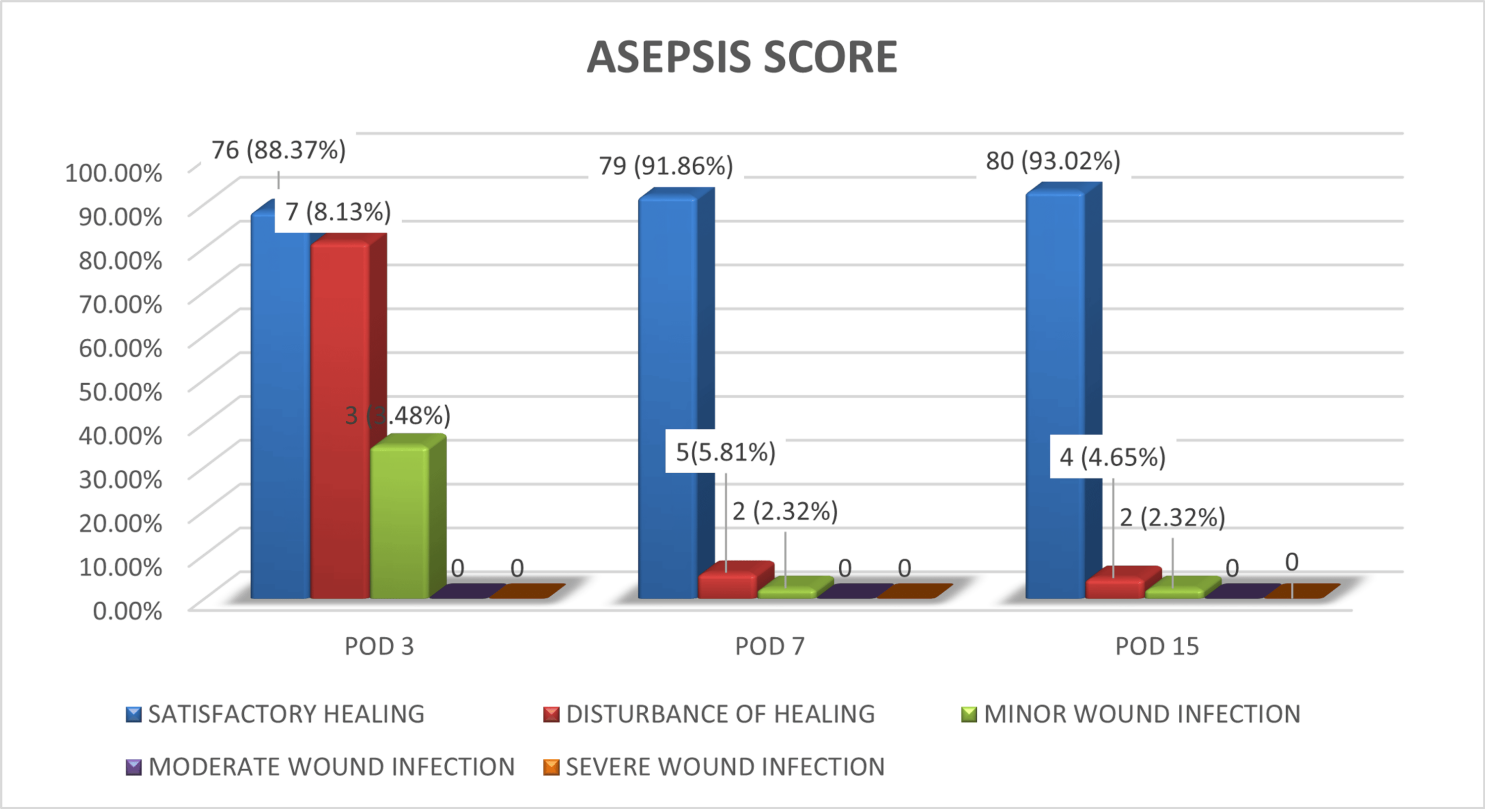
Distribution of ASEPSIS score on postoperative days 3, 7, and 15 (n = 86).

Surgical site healing was assessed using the ASEPSIS score. On POD three, 88.37% had satisfactory healing, improving further to 91.86% on POD seven, and 93.02% by POD 15. Minor wound infections were seen in three patients initially, decreasing over time. There were no cases of moderate or severe infection, indicating that postoperative infection control protocols were effective. The mean ASEPSIS scores dropped steadily from 25.75 on POD three to 8.95 by POD 15, further demonstrating effective wound management (Figure 2).

Hospital stay and readmission

**Figure 3:**
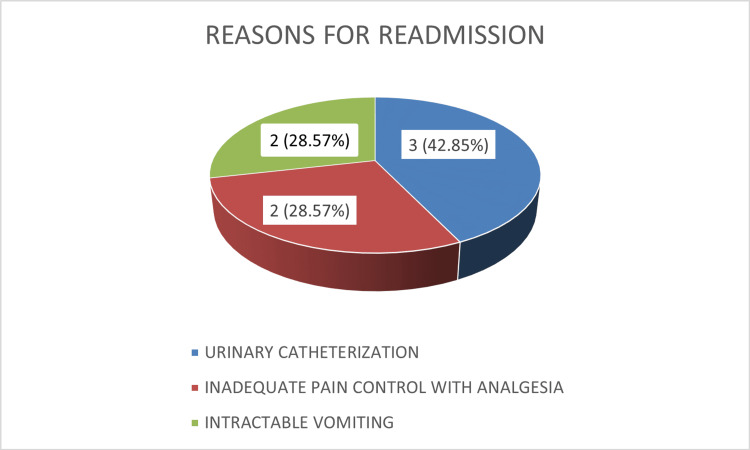
Distribution of reasons for readmission (n = 7).

The average hospital stay was 20.5 ± 2.4 hours, ranging from 4.5 to 23 hours, fulfilling the operational definition of day care surgery. However, seven (8.14%) patients required readmission. Reasons included urinary catheterization (42.85%), uncontrolled postoperative pain (28.57%), and intractable vomiting (28.57%). These causes reflect the most common challenges in ambulatory surgery, i.e., urinary retention, nausea, and inadequate analgesia, but remain within acceptable limits and were safely managed (Figure 3).

Patient satisfaction

Patient satisfaction was notably high, with 63.53% reporting being satisfied and 30.59% very satisfied. Only two patients were unsatisfied. This high satisfaction rate may be attributed to reduced hospitalization, fast recovery, good counseling, and the convenience of home-based recuperation. Likert-scale assessments provided qualitative evidence of psychological comfort and positive feedback, supporting the expansion of such models.

## Discussion

Day care surgery, which benefits from advancements in both surgical and anaesthetic techniques, has become a preferred mode of treatment in developed nations. However, in developing countries like India, its implementation faces challenges, including limited healthcare infrastructure, financial constraints in establishing dedicated units, and psychosocial factors influencing patient preference [1].

In the present study, the majority of patients were aged 31-40 years (38.82%), followed by 21-30 years (25.88%), with a mean age of 31.24 ± 11.54 years. Males constituted 72.94% of the cohort, a finding that correlates with other Indian studies. Lingaiah et al. reported 61.2% of males with a mean age of 45.6 years [9]. Hedawoo and Sheikh observed a younger mean age of 32.68 years and a male predominance of 94.7% [10]. Similarly, Vaishnav and Patel noted a male-to-female ratio of 57.69% to 42.31%, with a mean age of 41.70 years. Chowlu et al., however, reported a female majority (73%) with a mean age of 36.75 years [5,11].

Hernia repair emerged as the most frequent procedure in this study (20.93%). Globally, inguinal hernia repairs are widely done on a day-care basis, with rates as high as 90% in the United States, 80% in Denmark, and 75% in Sweden. However, in the present study, only 6.4% were laparoscopic repairs, reflecting low adoption rates in India compared to other nations [12,13]. This trend was echoed by Lingaiah et al., where 25.8% of day care procedures were hernia repairs, and by Hedawoo and Sheikh, who reported a 34.3% rate.

Other common procedures in this study included lipoma excision (10.46%) and lateral sphincterotomy (9.3%). Less common but notable surgeries were fibroadenoma excision, circumcision, laparoscopic appendectomy, cystoscopy, sebaceous cyst excision, and laparoscopic cholecystectomy. Studies by Lingaiah et al. and Hedawoo and Sheikh similarly reported a variety of general surgical and urological procedures, including appendectomy, hydrocele repair, hemorrhoidectomy, and excision biopsies.

Day care laparoscopic cholecystectomy (DCLC) is gaining global recognition for its safety, efficacy, and cost-effectiveness. While adoption rates are low in Europe (0.5-2%), the United States (49.8%) and Canada (43.9%) show much higher uptake [14]. In this study, 3.42% of patients underwent DCLC, all carefully selected based on criteria such as American Society of Anesthesiologists I/II status, short surgery duration, and no previous abdominal surgeries. Robinson et al. highlighted these criteria as predictors of success, while Ali et al. reported a 92% success rate in selected patients [15,16]. In contrast, Lingaiah et al., Vaishnav U and Patel, and Hedawoo and Sheikh did not report any DCLC cases, indicating a need for more structured implementation in India.

Pain management remains a significant factor influencing patient satisfaction. In this study, the mean postoperative pain score reduced from 5.04 to 0.58 within one day. Makwana et al. observed postoperative pain in 26% of patients, while Wig reported a 30-40% incidence of moderate-to-severe pain within 48 hours [17,18].

Postoperative nausea and vomiting was the most frequent complication (25.88%). This was higher than rates reported by Kala et al. (1.45%) and Ramyil et al. (0.72%), but comparable to Makwana et al. (15%) and Rashiq et al. (15-34%) [8,19,20]. The variance in PONV rates might be attributed to anesthetic agents used, patient profiles, and postoperative care standards.

Wound healing, as measured by the ASEPSIS score, improved steadily from POD 3 to 15. Surgical site infection was noted in seven (8.13%) patients, primarily in those who underwent abscess drainage or excision biopsies. This was slightly higher than infection rates reported by Lingaiah et al. (2.4%), Vaishnav and Patel (2.56%), but in line with findings reported by Pardhan et al. (7.7%) [21].

The mean hospital stay in this study was 20.5 ± 2.4 hours, ranging from 4.5 to 23 hours, closely aligning with durations reported by Lingaiah et al. (19.4 hours), Chowlu et al. (20.97 hours), and Phillips et al. (5-23 hours) [6]. This supports the feasibility of discharging patients within 24 hours with proper selection and postoperative protocols.

No mortality was reported, consistent with the findings of Lingaiah et al., Pota et al, Chowlu et al, and Vaishnav and Patel, reinforcing the low-risk nature of well-planned day care surgeries. The procedures performed under this model showed low morbidity and were well tolerated, affirming its potential as a standard of care.

This study has a few limitations. First, the follow-up period was relatively short, restricting the ability to assess long-term outcomes, recurrence rates, and delayed complications such as chronic pain or late infections. Second, the sample size was modest, which may limit the statistical power to detect rare complications or draw broader generalizations. A larger cohort might yield more robust conclusions and better subgroup analyses. Lastly, being a single-center study conducted at a tertiary care academic hospital, the findings may not be universally generalizable to other settings, particularly rural or resource-limited environments. Multi-center studies with longer follow-up durations and larger populations are warranted to validate and extend these findings. This study emphasizes the practical advantages of day care surgery in India’s resource-constrained environment. With proper planning, patient selection, and institutional support, this model can reduce hospital bed occupancy, lower healthcare costs, and improve patient satisfaction. For broader implementation, public awareness must increase, healthcare personnel must receive training, and insurance frameworks should cover day care procedures.

## Conclusions

This study reaffirms that day care surgery is a safe, effective, and patient-friendly alternative to traditional inpatient care. It offers substantial benefits, including reduced hospital stays, lower healthcare costs, faster recovery, and decreased risk of hospital-acquired infections, all the while maintaining high standards of clinical care. High patient satisfaction and minimal postoperative complications further validate its feasibility and acceptability. With appropriate patient selection, thorough preoperative evaluation, and structured postoperative follow-up, day care surgery can be effectively implemented across a wide range of surgical procedures. Its integration into mainstream surgical practice holds promise for optimizing healthcare resource utilization and advancing toward more patient-centered and cost-efficient healthcare delivery systems.
